# U. S. V. M. A.

**Published:** 1896-09

**Authors:** 


### SOCIETY PROCEEDINGS.

### U. S. V. M. A.

THE thirty-third annual convention of the Association was called to order at 10.15 A.M., at the University of Buffalo, by President W. Horace Hoskins, of Philadelphia. He at once introduced acting Mayor Boeckel, who welcomed the delegates to the "Queen City of the Lakes," and bid them to partake of her hospitality. Recognizing the importance of the work of the profession and their interest in sanitary measures for the welfare of the whole of our people, he heartily wished for the convention the greatest good from their deliberations that could flow from the gathering together of so many strong representatives, and assured one and all of the interest which would be taken in our work.

Vice-President Osgood feelingly responded to the Mayor and thanked him for the warm and generous character of his welcome, and strongly referred to the second great question which every city must solve for its people, the securing a pure milk-supply, and of its growing importance under the present strong light thrown upon it by the veterinary profession.

The President then delivered his annual address,[1] which was listened to with marked attention, and which hastily reviewed many aspects of the work of the profession, paying a warm tribute to the colleges and the members of the profession and rejoicing in the great advances made by those of our number in the great and unlimited sanitary field. To those conducting this work he had words of praise, and to the Chief of the Bureau of Animal Industry he paid just tribute to the worth of his trying and arduous work.

The roll-call during the several sessions noted the following members present: James L. Robertson, C. P. Lyman, D. J. Dixon, F. H. Osgood, W. L. Williams, J. F. Winchester, M. E. Knowles, William Dougherty, A. W. Clement, Thomas F. Barron, E. B. Ackerman, R. P. Lyman, W. Horace Hoskins, S. Stewart, A. F. Peters, L. Pope, Jr., D. E. Salmon, M. Stalker, C. A. Cary, N. P. Hinkley, G. E. Gangloff, S. Somerville, Jr., John Wende, C. J. Wilganz, Joseph Hughes, Thomas B. Rayner, James B. Rayner, Leonard Pearson, John R. Hart, M. R. Trumbower, H. D. Gill, W. H. Pendry, Roscoe R. Bell, George H. Berns, J. M. Wright, W. H. Dalrymple, W. C. Rayen, L. H. Howard, J. Payne Lowe, James McDonough, James Robertson, Joseph Hawkins, E. A. A. Grange, M. H. Reynolds, H. J. S. Weicksel, Wm. Henry Kelly, H. Sutterby, F. L. Kilborne, Wm. Jakeman, Frank H. Miller, S. J. Thompson, W. J. Hinman, E. H. Shepard, T. B. Cotton, Wilson Huff, S. Brenton, John A. Bell, B. P. Wende, Robert W. Ellis, Louis A. Robinson, H. S. Wende, W. E. Wight, H. M. Manley, W. L. Baker, Claude D. Morris.

Honorary member present, Prof. D. McEachran, Montreal, Canada.

As delegates from Pennsylvania: Drs. James T. McAnulty, J. C. Foelker, N. Rectenwald. From the National Master Horseshoers' Association, J. C. McKenzie, Rochester, N. Y.

[1] Will appear in the October number.

Among the visitors were Prof. A. Smith, Toronto, Canada; Prof. James Law, Ithaca, N. Y.; Drs. T. A. Crandell, Jr., Buffalo, N. Y.; M. W. Drake, Philadelphia; W. W. Gibbs, St. Thomas, Ontario; F. F. Hoffmann, Brookville, Pa.; R. Perkins, Hardys, New York; R. E. Collins, Memphis, Tenn.; John T. Claris, Buffalo, N. Y.; J. A. McCrank, C. J. Mulvey, Mark D. Williams, Middleport, N. Y.; Anderson Crowforth, Lockport, N. Y.; E. Rafter, Hamburg, N. Y.; Chas. Elliott, St. Catharine's, Ontario; Mr. Samuel N. Cook, of Business Men's League, Columbus, Ohio.

The *Comitia Minora* reported favorably upon the following list of applicants, and they were elected to membership on information from the Secretary that they had complied with the requirements:

Prof. James Law, M.R.C.V.S., Ithaca, N. Y.; W. C. Rayen, D.V.S., Nashville, Tenn.; Charles Higgins, D.V.S., Dover, Mass.; E. P. Shaffter, V. S., Kansas City, Mo.; H. Wellner, D.V.S., Providence, R. I.; F. H. Miller, V.S., Burlington, Vt.; F. W. Hopkins, D.V.S., Kansas City, Mo.; A. S. Wheeler, V.M.D., New Orleans, La.; W. H. Ridge, V.M.D., Trevose, Pa.; W. H. Kelly, V.S., Albany, N. Y.; S. J. Thompson, V.S., Winnipeg, Manitoba; W. J. Hinman, V.S., Winnipeg, Manitoba; T. B. Cotton, V.S., Mt. Vernon, O.; S. B. Staples, D.V.S., Baton Rouge, La.; E. H. Shepard, V.S., Cleveland, O.; F. B. Tucker, D.V.S., Elizabeth, N. J.; J. P. O'Leary, M.D.V., East Buffalo, N. Y.; W. H. Gribble, D.V.S., Washington C. H., O.; H. M. Manley, V.S., Miamisburg, O.; H. S. Wende, V.S., Tonawanda, N. Y.; W. E. Wight, V.S., Delaware, O.; E. L. Quitman, M.D.C., Chicago, Ill.

The application of Alex. Crowforth, V.S., Lockport, N. Y., was refused. The application of G. W. Vernon, of Missouri, was withdrawn. C. A. Keene, Fitchburg, Mass., refused.

A number of resignations were accepted, the Secretary reporting that they had complied with all the requirements, and their dues were paid to date.

It was recommended and adopted to change the name *Comitia Minora* to Executive Committee.

The recommendation to allow the Executive Committee to fix the date of the annual meeting was not approved, owing to the confusion it would lead to in paying dues and arranging members' business to attend the meetings.

Owing to insufficient knowledge, the Executive Committee did not indorse the application for honorary membership of Veterinarian Desmond, Warnamboul, Australia.

The several amendments offered, to fix a definite allowance for veterinary colleges, to allow graduates of agricultural colleges, and to strike from the lists of recognized colleges those that made false statements in their catalogues, were not approved by the Executive Committee, whose action was sustained by the Association.

It was, on recommendation of the Executive Committee, approved that all reports and papers, where the chairmen or writers were absent, that said reports and papers go to the foot of the programme.

The resignation of Dr. S. K. Johnson, of New York City, was refused, pending action of the New York County Veterinary Medical Association, before whom charges of violation of the code of ethics were pending.

Protest filed by Dr. R. A. Archibald, who claimed to have been unfairly and unjustly discharged by the Bureau of Animal Industry, was examined into, and it was learned that his appointment was made for a probationary period of six months, and that at the expiration of this time, on the failure of his superior officers to recommend his retention, he was discharged in accordance with the rules of the Department, and there was no cause for action upon the part of the Association.

The Army Legislative Committee made a report of its work and the progress made, the present favorable position of the bill, and its sure promise of success at the coming Congress. Their request for funds was well received and its concession left to the officers, who were empowered to so award if the treasury would permit. The old chairman, Dr. J. P. Turner, was continued by the newly elected President, with Dr. John R. Hart and Dr. Austin Peters added to complete the committee.

The Committee on Publication, through the chairman, Dr. W. L. Williams, made a very complete and satisfactory report, and in closing made a number of recommendations to the Association as to the best plans for control of the reports, papers, and proceedings, and to obtain reprints of the same for the members. The report exhibited the most earnest and thorough consideration at the hands of the committee, and, after some discussion and advice from those representing the several veterinary publications, it was, on motion, moved to refer the whole matter back to the Publication Committee with power to act. President-elect Osgood retained by announcement the old committee, with Dr. W. L. Williams as chairman, and Messrs. Hinkley and Stewart as his aids.

All the members of the Committee on Act of Incorporation being absent, the President announced that nothing had been done during the year owing to a depleted treasury and also to the fact of the disposition of the President to not look upon such acts with favor, having declined to approve a similar act proposed for the National Florists' Association. It was therefore deemed unwise to press the matter, owing to the fact that Congress exhibited a marked indifference to entering upon any new legislative enactments in view of the coming national election. This course carried with it the proposition to adopt a seal or emblem, as the latter could not be controlled unless we had a national act of incorporation.

The Finance Committee was not able to make a complete report at this time, owing to its inability to secure the full report of the last committee, and a large number of bills approved and unpaid, but was able to assure the Association of the better financial condition and the ability to discharge all past indebtedness and to have sufficient money to publish promptly the proceedings of this year, and to deliver the same to all the members at an early date; also that a full and complete report would appear in the *Transactions* of the Association.

State Secretaries Cary, of Alabama, R. P. Lyman, of Connecticut, W. H. Dalrymple, of Louisiana, S. Brenton, of Michigan, M. H. Reynolds, of Minnesota, W. L. Williams, of Montana, A. T. Peters, of Nebraska, J. P. Lowe, of New Jersey, Lemuel Pope, Jr., of New Hampshire, reported in person for their several States, and many reports were filed with the Secretary and referred to the Publication Committee. Dr. Lyman, of Connecticut, made special reference to the stamping out of bovine tuberculosis in the Nutmeg State and of the hearty co-operation of the farmers and dairymen. Dr. Lowe made special reference to the need of a strong single association in New Jersey and of better legislation needed. He reported much more active work by city and local boards of health, and that they were thoroughly alive to the good work being done elsewhere.

The Secretary's report was very brief and extremely modest in its review of the work done by that officer during the past year. As the work of the convention proceeded and the general standing of Association affairs became better known, it became more evident to all how efficiently and zealously Secretary Stewart had worked, and his unanimous recall to this post of duty was a well earned recognition of his fidelity to the Association's interests. President Osgood will have an efficient aid at his side during the coming year, and we are sure an equally favorable report will be forthcoming in 1897.

The President then called on Dr. H. D. Gill to present his paper on "The Relation of the Veterinarian and Veterinary Colleges to Master Horseshoers." This proved a topic of the greatest interest and provoked the widest variance of opinion. This paper was opened for discussion by Drs. James Robertson, of Chicago, J. C. McKenzie, of Rochester, N. Y., and James T. McAnulty, of Philadelphia, representing the Committee on Science and Education of the National Master Horseshoers' Association. Each of them in turn voiced the appeal for assistance from the U. S. V. M. A. in well-directed remarks and earnest solicitude for the welfare of their craft, forcibly recalling the disadvantages under which they had long struggled and, thoroughly aroused to the needs of the day, they won the marked attention of all.

Dr. Joseph Hughes, having been appointed a speaker upon the subject, opened the subject for discussion and outlined his work in connection with instruction of shoers at Chicago. He believed that normal shoeing, preservation of the sound foot, anatomy, physiology of the foot, and locomotion should be taught; but the line should be drawn at pathological shoeing as the domain of the veterinarian. He was followed by Dr. Leonard Pearson, who dwelt upon some of the dangers in establishing schools of farriery, and how important it would be to do this work well if it was going to be of real value. Dr. Jos. Hawkins, of Detroit, condemned severely the methods pursued at Detroit in this direction and the results that had followed in that city, resulting in much clashing of the veterinary profession with members of the shoeing craft so schooled. Dr. Dixon, of Hoboken, N. J., was opposed to any recognition of the craft or the attempt of veterinarians to teach them. He did not consider it necessary for them to have a knowledge of anatomy, physiology, or locomotion, but said it was a matter of handicraft and could not be taught by veterinarians. Dr. McDonough, of Montclair, N. J., an old shoer, in a very humorous vein said that in a few years there would not be any of them capable of making a shoe, as almost all shoes were now machine-made. As for pathological shoes they had to be made, could not be bought, and it required skill of a high character to determine what was needed, then to make it and properly apply it.

(To be continued.)

